# Medical Fraternalism and Medical Citizenship*Read before the Parker-Palo Pinto County Medical Society at Weatherford, Texas, February 13, 1912.

**Published:** 1912-07

**Authors:** Alexander S. Garrett

**Affiliations:** Springtown, Texas


### TEXAS MEDICAL JOURNAL.

Established July, 1885

F. E. DANIEL, M. D., - - - - Editor, Publisher and Proprietor

Published Monthly.—Subscription, $1.00 a Year.

VOL. XXVIII. AUSTIN, JULY, 1912. No. 1.

The publisher is not responsible for the views of the contributors.

### Original Articles.

For Texas Medical Journal.

### Medical Fraternalism and Medical Citizenship.*

BY ALEXANDER S. GARRETT, M. D., SPRINGTOWN, TEXAS.

The term "Medical Fraternity" conveys to the mind the thought of "brotherhood," and a common interest and a fellow-feeling for the welfare, good will, fortune and happiness of each other.

The prevailing influence of this sentiment prompted the writing of a code of Medical Ethics, which is but an expansion or amplification of the Golden Rule laid down by the Great Master Physician Himself. The pleasant relationship, the brotherly love and sympathetic care that exists, or should exist, between all broad-minded physicians ought not to be excelled by any other profession, the ministry not excepted.

The humanitarian nature of our profession—that of treating diseases, relieving suffering humanity, trying to prevent sickness and save human life—is of a nature disposed to promote and develop a kindness of heart and a fraternal spirit.

The true fraternal spirit is both exemplified and experienced when a brother physician or some member of his family falls sick, or is the unfortunate victim of some serious accident; then and there the spirit of the blessed Master is both experienced and observed as a practical working force in all its fullness.

Among all true physicians the big "I" and little "you" does not prevail or manifest itself. Perchance, nature or circumstances, favorable opportunities and environment, one or all these

*Read before the Parker-Palo Pinto County Medical Society at Weatherford, Texas, February 13, 1912.

conditions may have contributed to the success and prominence of one physician more than another, as is often the case, but it never makes the true physician act in an egotistical manner in the presence of his less fortunate brother. "Charity vaunteth not itself; is not puffed up."

Our inability to stay the hand of death when the young, the lovely, the intellectual, the good, the bad, the rich and the poor, all classes, are seized in death's unyielding grip, and anxious and praying hearts look to us to break his hold, and we look on as weak as infants in his presence, is enough to remove the vain conceit from the head of the most self-important tyro of the profession.

Yes, my brother physicians, ofttimes we are made to realize that our best efforts are futile, and, if help comes at all, it must come from the inexhaustible source above, from whence comes all our blessings, and which blessings it may be often comes in answer to sincere and earnest prayer.

The fraternal spirit should be so strong among physicians that quarrels, contentions and jealousies should never arise among them, and the effort of one physician to seek by questionable or unethical methods to obtain possession of another physician's practice, whether it be a single case or many, should be considered an anomaly of an unusual and a serious nature from a professional standpoint.

In the consultation room the character of the physician and his fraternal relations toward his brother physicians or physician is often disclosed. Here is the place, as much if not more than any other, where the physician must be on his guard. The eyes and ears of all present are open to see and hear what the consultant does and says in regard to the case. Shame on the physician who, when called in to assist his brother physician in trying to cure the sick, will seek to stab him and steal his patronage and influence from him. Instead of it being said, "Behold how they fight each other," it should be always said of physicians closely associated in towns and cities, "Behold how they love each other and how they work together in unity and harmony for the good of humanity."

Physicians should be leaders and lights to the community and State in every good and noble work. How careful, and how anxious, and how eager the mother listens to get the directions correct, and to hear what the "doctor" has to say! This only illustrates the powerful influence that is wielded by every physician, either consciously or unconsciously, who practices his profession.

For thousands of years the physician and priest were one and the same. While in the present age the two professions are separated, no priest or preacher should excel the physician in its fullest sense in moral influence in both private and public life.

The famous Hippocratic oath contains the following sentences, which denote the high moral standard required of physicians in those ancient days: "I will give no deadly medicine to man, woman or child born or unborn. With purity and with holiness I will pass my life and practice my art."

Jesus Christ, whose name is honored in all the world, and who is believed by many millions of people to be the world's Redeemer and the Savior of the souls of men, was a physician, and His greatest work of which we have any knowledge was as a traveling doctor. If we have a correct history of His wonderful life, He was truly a Divine Healer, and received His commission to practice direct from God. When He commissioned His twelve disciples to go forth into all the world and preach, to "heal the sick" was one of the first commands contained in the Divine commission. A large part of the history of Christ, His life and work, and the history of the early church, is said to have been written by Luke, the "beloved physician." It is claimed by writers on foreign missionary work, as it is termed in regard to preaching the gospel of Christ, that the most effective work and the greatest opening into heathen countries for the Christian religion has been accomplished by and through medical missionaries.

Whether the physician should be both preacher and physician at the present day, as he was in ages past, is not for me to say, but that he should be a man of the highest and purest type of morals, and a leader in all that pertains to the uplifting and making better man's social, intellectual and moral conditions, with special leadership in matters that pertain to public health and hygiene, I have not the remotest doubt.

As Christ, the mighty Son of God,
In love the sick did heal,
So we, who follow in His steps,
Should live, and act and feel.

No envies in our hearts should be,
No unkind words be said
But peace, love and unity
By all the world be read.

Thus as one man our lives should be,
With aims and hopes sublime,
The sick to heal, the slaves to free,
And love to show to all mankind.

Of so great renown was I-em-hotep, the first physician of whom we have any definite knowledge or special history, who lived three thousand and five hundred years B. C., that after his death he was deified as the God of Medicine. He is said to have been a physician, minister to the king, architect, alchemist, and astronomer.

The opportunities of the physician to serve humanity are numerous and great. And every true physician, so far as the circumstances and opportunities will permit, ought to be in touch and in sympathy with every onward, upward, intellectual, moral, uplifting and sanitary movement of the community, State and Nation.

It is needless to say that no true physician or surgeon ever encourages unnecessary treatment or seeks to prolong cases, makes unnecessary visits or advises operations simply for the purpose of increasing his bank account. Of the physician, it can be truly said that "He sweareth to his own hurt," for he is found in the lead searching out the causes of diseases, giving advice in hygiene, in spreading a knowledge of preventive medicine, by and through which means the very source of his income is made less.

Preventive medicine is the highest branch in medical science, and is one in which modern physicians are vying with each other to excel. It is through their influence as leaders combined with the influence of other humanitarians that the world is to be relieved of its ignorance concerning diseases, and that human suffering is to be abolished and man restored to his primeval greatness from a standpoint of health, and Paradise regained on earth.

To reach out and accomplish all these great and important things that physicians must do, and that devolves upon them as good citizens to do, some of the profession must launch out, as of old, into the ministry, some into other professions, and some into politics, and so mix and mingle with all public life that the very best thoughts and knowledge that they possess shall permeate the universe and become common knowledge, so that all the people may know, as much as it is possible to know, how to escape sickness and suffering, and how to so improve and develop the human race that men and women can and will be truly called the sons and daughters of God.

So long as men and women are dying from the effects of alcohol, crime is committed under its influence, women are made widows, and children are made orphans because of alcohol, poverty, ignorance, immorality, and shame result from its use; so long as venereal diseases are playing havoc with the very vitality of our nation, and are sowing their seeds of poison and destruction broadcast over the land; so long as infants die on account of the ignorance of their parents; so long as tuberculosis mows down hundreds and thousands of our country's best blood every year; so long as ignorance, sin, sickness, suffering and premature deaths sway in ceaseless activity from the center to the circumference of our land, so long will there be a great work for physicians in helping to stay the powers of sin and darkness, and in bringing to light, love, liberty and universal brotherhood to mankind. The field of the physician is large, and his work is very great. They want to do much, and they are destined to do much for humanity.

In order to accomplish greater results by and through the medical profession, our leaders in the dissemination of a knowledge of preventive medicine favor the establishment of a Department of Public Health. This, they think, will be a forward movement in national health legislation that will be of inestimable value to the nation. With this noble and worthy object in view, the medical profession should stand in one unbroken band in favor of and in support of such legislation.

